# Response to: Comment on “Are Patients with Polycystic Ovarian Syndrome Ideal Candidates for Oocyte Donation?”

**DOI:** 10.1155/2017/1467136

**Published:** 2017-09-18

**Authors:** George Queiroz Vaz, Alessandra Viviane Evangelista, Cassio Alessandro Paganoti Sartorio, Maria Cecilia Almeida Cardoso, Maria Cecilia Erthal, Paulo Gallo, Marco Aurelio Pinho Oliveira

**Affiliations:** ^1^Department of Gynecology, Rio de Janeiro State University, 20551-030 Rio de Janeiro, RJ, Brazil; ^2^Vida Fertility Center, 22793-080 Rio de Janeiro, RJ, Brazil

Chetan and Dada commented on our study [1] titled “Are Patients with Polycystic Ovarian Syndrome Ideal Candidates for Oocyte Donation?” that analyzed the impact of PCOS in oocytes donation treatments [2].

The role of donated oocytes in the treatment of infertile women, especially in advanced age, is well established.

However, there are different practices for obtaining donated oocytes [3]. It is worth noting that in Brazil, due to local law, only oocytes from patients who are undergoing in vitro fertilization and who decide to donate them altruistically can be used [4]. In addition to concerns regarding the age of the donor, we should note the infertility that led the donor to have the indication of IVF and consider whether this will influence the outcome of the pregnancy in the recipient.

Chetan and Dada drew attention to the importance of male factors in the success of implantation and in the clinical pregnancy rate [1], especially the paternal effect of sperm DNA damage in the stages of embryonic development, and also criticized the fact that the male factors have not been studied in this study. Obviously, we agree on the importance of male factors in IVF success rates. The objective of our study was to know if there would be any impact on the IVF results of recipient patients with donor oocytes from infertile women with a diagnosis of PCOS compared to donors with no PCOS. In our study, there is no reason to believe that male factors would be unbalanced between those two groups of recipients. Male factors probably influenced negatively the results of both groups of recipients.

At the end of their letter to the editor, Chetan and Dada looked at the altered hormonal environment in women with PCOS. They raised a concern that it could affect the oocyte epigenome, in addition to these oocytes negatively affecting fertilization, implantation, and pregnancy rates. We had the same concerns, which motivated us to delineate this clinical study.

A meta-analysis by Heijnen et al. includes nine studies comparing reproductive outcomes of IVF in patients with PCOS and no PCOS [5]. They did not find any difference in pregnancy rates between the groups. Similarly, in our study, we did not find differences in clinical pregnancy rates in oocyte recipients patients with PCOS compared to non-PCOS patients. We understand Chetan and Dada's claim that oocytes from patients with PCOS should be used with caution, but it seems from our results that patients with polycystic ovary syndrome should not be discarded for oocyte donation programs [2].
